# Multiple organ injury in septic rats under weightlessness

**DOI:** 10.3389/fphys.2026.1849176

**Published:** 2026-07-17

**Authors:** Rui Zhai, Jie Gao, Ye Tian, Lijun Zhao, Lei Zhu, Jianwen Gu, Mei Hu

**Affiliations:** 1 Department of Critical Care Medicine, The Ninth Medical Center of Chinese People’s Liberation Army General Hospital, Beijing, China; 2 Research Department, The Ninth Medical Center of Chinese People’s Liberation Army General Hospital, Beijing, China; 3 Key Laboratory of Environmental Sense Organ Stess and Health, Ministry of Ecology and Environment, Beijing, China; 4 Graduate School, Anhui Medical University, Hefei, China; 5 School of Clinical Medicine, Qinghai University, Xining, China

**Keywords:** coagulation dysfunction, infection, inflammatory storm, multiple organ injury, sepsis, weightlessness

## Abstract

**Introduction:**

As human space exploration advances, the risk of infection faced by astronauts has also become apparent. However, the impact of sepsis on the body under weightlessness remains unclear.

**Methods:**

A rat model of sepsis under simulated weightlessness was preliminarily established by combining hindlimb unloading with cecal ligation and puncture. The effects of simulated weightlessness on the early progression of sepsis in rats were systematically evaluated through assessments of mortality, hematological parameters, inflammatory markers, coagulation function, organ function, and histopathological injury.

**Results:**

Our results demonstrated that simulated weightlessness reduced the survival rate of septic rats. During the early phase of sepsis, compared with septic rats under normal conditions, those under simulated weightlessness showed significantly upregulated levels of MON, GRA, TNF-α, IL-6, and CRP, and significantly downregulated IL-10, suggesting that weightlessness aggravated the inflammatory storm in septic rats. Meanwhile, in septic rats under simulated weightlessness, PLT was significantly decreased, while PT, APTT, and FIB were significantly increased, indicating that weightlessness aggravated early coagulation dysfunction in septic rats. Furthermore, the significant increases in ALT, AST, BUN, CRER, CK, and LDH, combined with histopathological findings from HE staining of organ sections, suggested that weightlessness aggravated early multiple organ injury (heart, liver, lung, and kidney) in septic rats.

**Discussion:**

These findings highlight the necessity of proactive environmental monitoring and rigorous preventive measures for astronauts during space missions. While understanding sepsis mechanisms in weightlessness is vital for emergency preparedness, the precise molecular pathways and effective treatments remain unclear, thus warranting further investigation.

## 1 Introduction

Camilla Urbaniak et al. profiled the microbial communities on eight surfaces of the International Space Station in a 14-month study starting in 2022;antimicrobial resistance genes were detected in all samples, with resistance to macrolides, lincosamides, and streptogramins being notably prevalent (Urbaniak et al., 2022). In 2025, Megan S. Hill et al. reported that a novel bacterial strain named 1F8SW-P5T, isolated from the crew cabin wall of the International Space Station, showed an increase in relative abundance over an 8-year period and demonstrated resistance to fluoroquinolone antibiotics, two types of β-lactam antibiotics, and one macrolide antibiotic (Hill et al., 2025). These drug-resistant and unknown microorganisms indicate that microbes on the inner surfaces and equipment of space habitats pose a potential threat to human health. Furthermore, various factors in the spaceflight environment may weaken the immune systems of crew members engaged in space missions, thereby increasing the risk of infection during space travel (Klaus and Howard, 2006). Given the inability to completely prevent infections in astronauts within the weightlessness environment of spacecraft, it is crucial to investigate the pathogenesis and therapeutic approaches for post-weightlessness infections, particularly those leading to sepsis. Tesfaye Belay et al. (2002) used a hindlimb unloading model to simulate the effects of spaceflight on mice’s resistance to Klebsiella pneumoniae infection (Belay et al., 2002), and Hernan Aviles et al. (2003) reported increased susceptibility to Pseudomonas aeruginosa infection under hindlimb unloading conditions (Aviles et al., 2003). However, a single pathogen infection has a short time course and is likely to activate only a single immune pathway, making it insufficient to reflect the complex progression of sepsis (Busch et al., 2020). Moreover, the pathogenic bacteria in the spaceflight environment remain unknown and diverse, necessitating further exploration of new disease models. LPS-induced sepsis is predominantly characterized by acute inflammatory injury, generally lacking immunosuppression and organ failure, and thus fails to mimic the clinical reality (Ling et al., 2024; Gong et al., 2026). In this study, we combined the hindlimb unloading (HU) model with the cecal ligation and puncture (CLP) model to establish a novel rat model of sepsis under simulated weightlessness. This combination captures the alterations in the gut microbiota under weightlessness, allows for opportunistic infection by susceptible flora in the weightless state, triggers the release of PAMPs, and encompasses the complete progression from local infection to bacteremia and subsequently to systemic sepsis (Su et al., 2023; Ling et al., 2024). We hope this work will lay the foundation for investigating the mechanisms of infection in a weightless environment.

## 2 Methods

### 2.1 Animal

144 male Sprague-Dawley (SD) rats, aged 6 -8 weeks, specific pathogen-free (SPF), were purchased from Beijing Vital River Laboratory Animal Technology Co., Ltd. (Certificate No.: SYXK (Jing) 2022-0052). After acquisition, the rats were acclimated for seven days in the animal facility. The rats were randomly and equally divided into 6 groups, namely the control group, hindlimb unloading (HU) group, sham group, cecal ligation and puncture (CLP) group, HU + sham group, and HU + CLP group. HU group: Rats were suspended by the tail continuously and placed in a 30° head-down tilt position relative to the horizontal plane for 28 days, with free access to food and water. Sham group: Rats were housed under normal conditions for 28 days, then underwent a sham procedure under aseptic conditions and isoflurane anesthesia. CLP group: Rats were housed under normal conditions for 28 days. Under aseptic conditions and isoflurane anesthesia, a model with higher severity was selected (Rittirsch et al., 2009): 75% of the distal cecum was ligated, and the ligated portion was punctured with a sterile 21-gauge needle. HU + sham group: After 28 days of HU, rats underwent a sham procedure under aseptic conditions and isoflurane anesthesia. HU + CLP group: After 28 days of HU, under aseptic conditions and isoflurane anesthesia, 75% of the distal cecum was ligated and the ligated portion was punctured with a sterile 21-gauge needle. Considering that spaceflight involves continuous exposure to weightlessness, both the HU + sham group and the HU + CLP group were returned to HU after postoperative recovery (within 3 hours). In each group, 15 rats were continuously monitored for 7-day survival rates after the procedure. At 24 hours post−procedure, 6 rats from each group were randomly selected for abdominal aorta blood collection under isoflurane anesthesia, followed by euthanasia. The anesthesia procedure was as follows: 3.0%–5.0% isoflurane was used for induction, after which the concentration was reduced to 1.5%–3.0% for maintenance anesthesia. All animal experimentation protocols were approved by the Animal Ethics Committee of the PLA General Hospital (Approval No.: LL-LCSY-2025-16).

### 2.2 Blood tests

Rat plasma was anticoagulated with EDTA, and complete blood count analysis was performed using the HF-3800 analyzer (HLIFE, China) according to the manufacturer’s standard operating procedures. For coagulation tests, plasma anticoagulated with sodium citrate was analyzed using the PUN-204813 system (Pulang, China) in accordance with the manufacturer’s instructions.

### 2.3 Inflammatory cytokines

Blood samples were collected from rats and allowed to stand at room temperature for 60 minutes. After centrifugation at 3,000 rpm for 15 min at 4 °C, the supernatants were collected. TNF−α, IL−1β, CRP, and PCT concentrations were determined by ELISA using commercial kits (cat. nos. MD120490, MD132589, MD3125, and MD5263, MDL, China) according to the manufacturer’s protocols.

### 2.4 Serum organ damage biomarkers

Levels of alanine aminotransferase (ALT), aspartate aminotransferase (AST), total protein (TP), albumin (ALB), blood urea nitrogen (BUN), creatinine (CREA), lactate dehydrogenase (LDH), and creatine kinase (CK) in serum were measured using an automated analyzer (XR200PLUS, Xinrui, China) according to the manufacturer’s instructions.

### 2.5 Pathological staining

After dissecting the heart, lung, and kidney tissues from the rats, the tissues were fixed in 4% paraformaldehyde at room temperature for 48 hours, dehydrated through a graded ethanol series, and cleared twice in xylene. After three changes of paraffin for infiltration, the tissues were embedded into paraffin blocks, sectioned at a thickness of 4 μm, and then deparaffinized, rehydrated, and stained with hematoxylin and eosin (H&E). The slides were examined using a fully automatic scanner (KF-PRO-005, KFBIO, China). Five random fields of view were selected per slide for semi-quantitative analysis. Lung tissue was scored from 0 to 4 for each of the following three aspects (mild to severe): inflammatory infiltration, alveolar wall thickening, and alveolar hemorrhage. Heart tissue was scored from 0 to 4 for each of the following three aspects: inflammatory infiltration, myocardial fiber necrosis/lysis, and edema/hemorrhage. Kidney tissue was scored from 0 to 4 for each of the following three aspects: inflammatory infiltration, glomerular/tubular injury, and edema/hemorrhage. For all scores, 0 represents the mildest and 12 represents the most severe.

### 2.6 Statistical analysis

Experimental data were analyzed using GraphPad Prism 9 software and are expressed as mean ± Standard Error of the Mean (mean ± SEM). For comparisons among three or more groups, one-way analysis of variance (one-way ANOVA) was used. Dunnett’s or Tukey’s method was used for pairwise comparisons among multiple groups. *P* value < 0.05 was considered statistically significant.

## 3 Result

### 3.1 Establishment of a sepsis rat model under simulated weightlessness conditions

**Figure 1:**
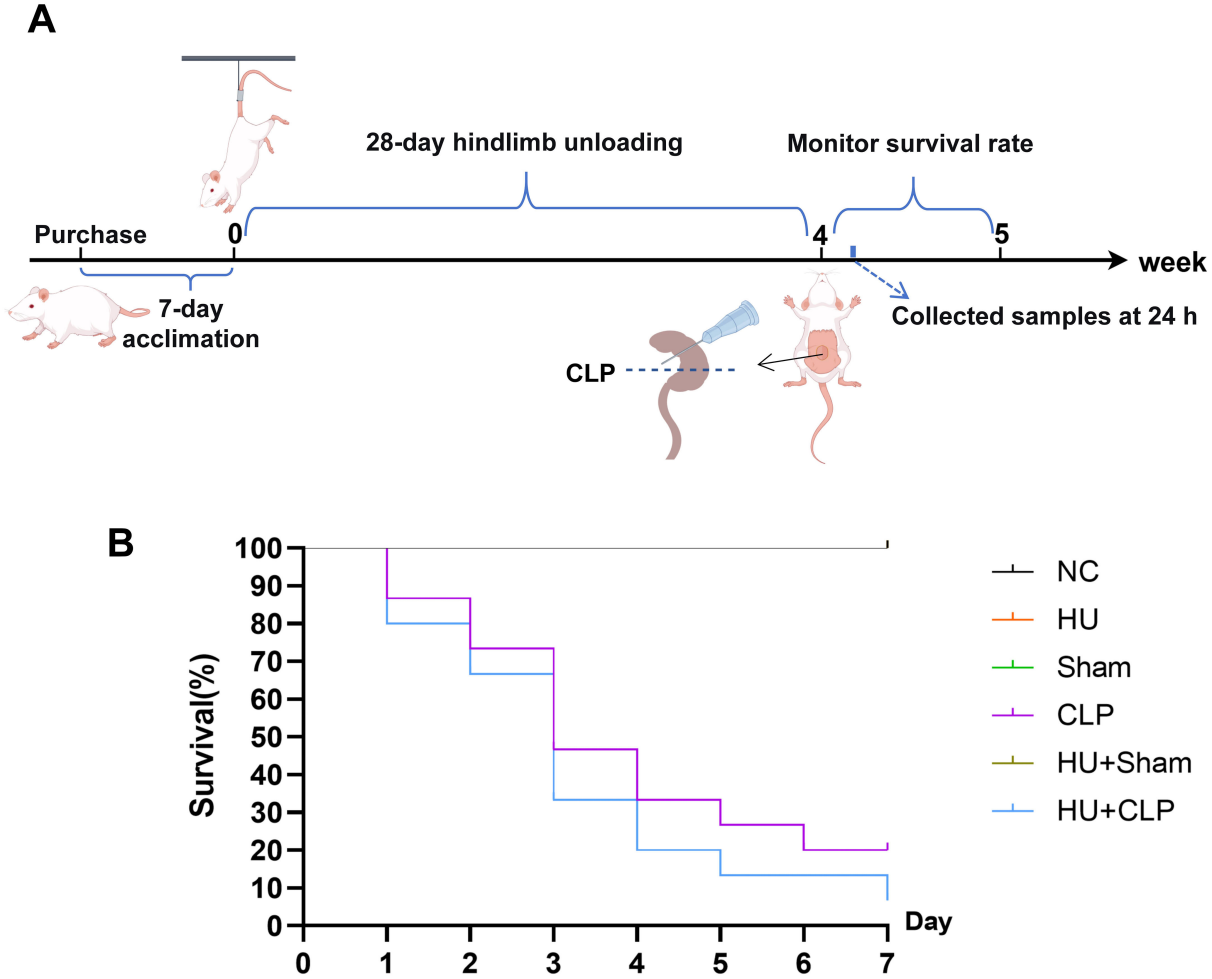
Experimental workflow and survival curves. **(A)** Experimental flowchart. **(B)** Seven-day survival curve of weightlessness sepsis rats, N = 15.

To further understand the effects of sepsis on the body under weightlessness, we established a weightlessness sepsis rat model (**Figure 1A**). As shown in **Figure 1B**, within 7 days, the survival rates of rats in the NC, HU, Sham, and HU+Sham groups were 100%, while it was 20% in the CLP group and 6.67% in the HU+CLP group. Weightlessness decreased the survival rate of septic rats.

### 3.2 Simulated weightlessness exacerbated the inflammatory storm in septic rats

**Figure 2:**
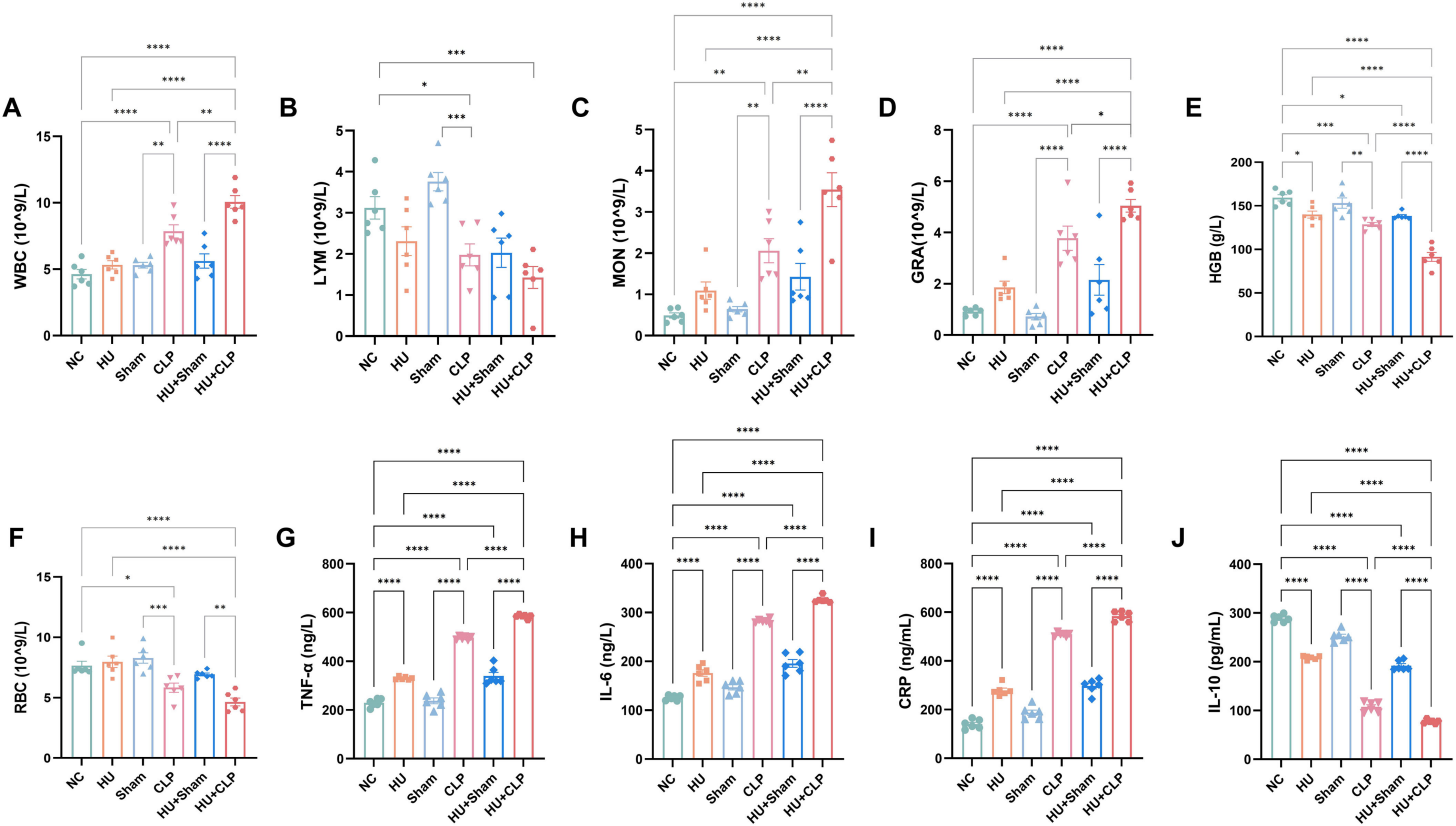
Detection of inflammatory cells and related inflammatory factors in septic rats under weightlessness conditions. **(A)** White blood cells; **(B)** Lymphocytes; **(C)** Monocytes; **(D)** Granulocytes; **(E)** Hemoglobin; **(F)** Red blood cells; **(G)** Tumor Necrosis Factor_alpha; **(H)** Interleukin_6; **(I)** C_reactive protein; **(J)** Interleukin_10. *P < 0.05, **P < 0.01, ***P < 0.001, ****P < 0.0001, N = 6.

Blood samples were collected 24 hours after surgery for routine hematological tests. Under weightless conditions, rat white blood cells, lymphocytes, monocytes, and granulocytes showed certain trends of change, but without statistical significance (**Figures 2A–D**). Twenty-four hours after CLP surgery, the white blood cell count in rats (**Figure 2A**) increased significantly, accompanied by a marked decrease in lymphocytes (**Figure 2B**), while monocytes (**Figure 2C**) and granulocytes (**Figure 2D**) showed significant increases. Compared with the CLP group under normal conditions, the white blood cells in the HU+CLP group were significantly upregulated; specifically, compared with the CLP group, the monocytes (**Figure 2C**) and granulocytes (**Figure 2D**) in the HU+CLP group were significantly upregulated. Weightlessness exacerbated early immune dysregulation in septic rats. Under septic conditions, the hemoglobin and red blood cell counts in rats significantly decreased (**Figures 2E, F**). Compared with the CLP group, the HGB level in the HU+CLP group was significantly decreased (**Figure 2E**).

We further assessed inflammatory cytokine levels in rats by ELISA. The results demonstrated that exposure to HU or CLP alone significantly increased the pro−inflammatory cytokines TNF−α, IL−6, and CRP (**Figure 2G–I**), while decreasing the anti−inflammatory cytokine IL−10 (**Figure 2J**), suggestive of an inflammatory response. Moreover, the HU + CLP group showed more pronounced elevations in TNF−α, IL−6, and CRP and a greater reduction in IL−10 compared with the HU alone or CLP alone groups. Taken together, these alterations in hematological and inflammatory markers suggest that weightless conditions aggravate the inflammatory storm in septic rats.

### 3.3 Simulated weightlessness exacerbated coagulation dysfunction in septic rats

**Figure 3:**
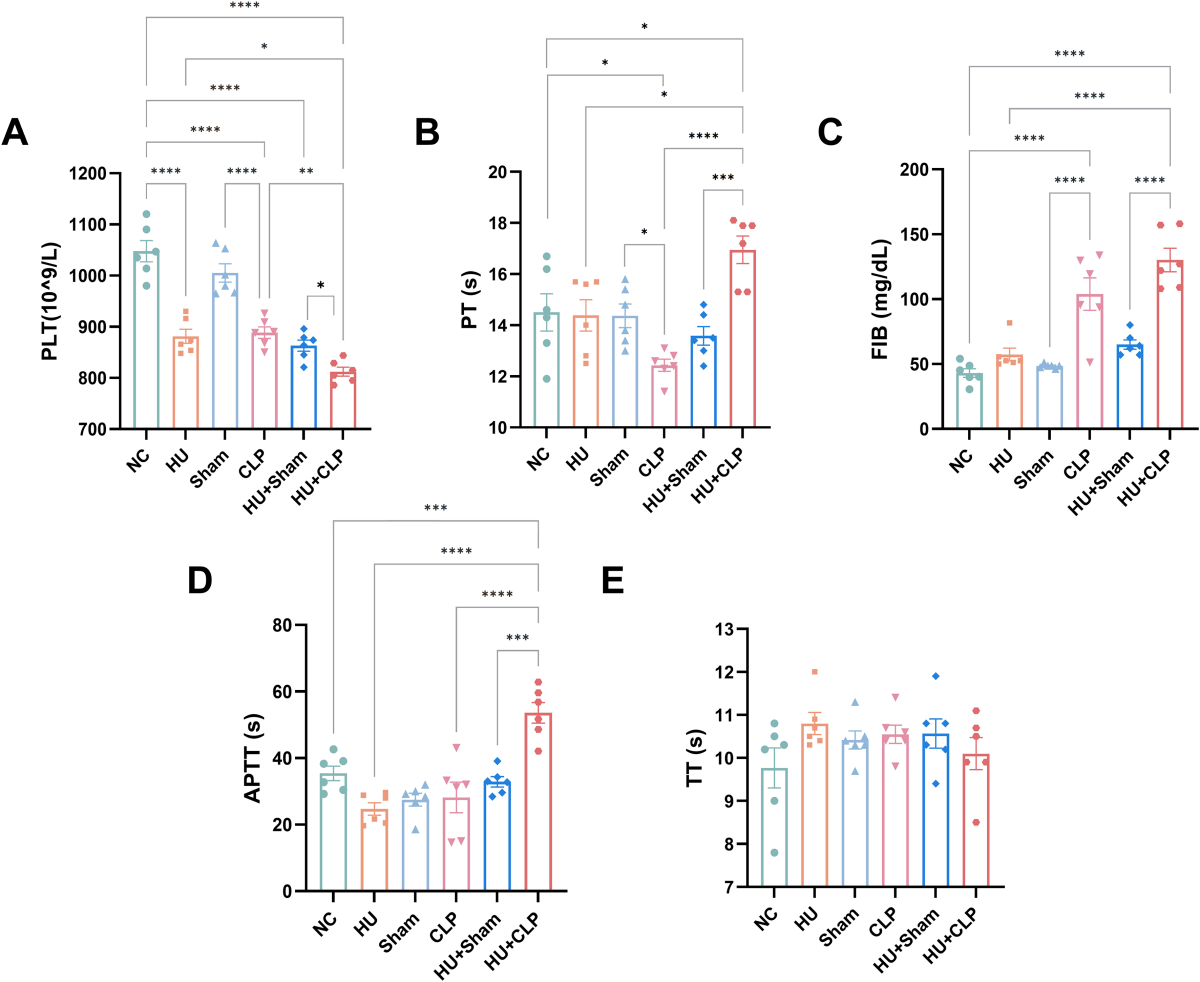
Coagulation function tests in weightlessness-simulated septic rats. **(A)** platelet count; **(B)** Activated partial thromboplastin time; **(C)** Prothrombin time; **(D)** Thrombin time; **(E)** Fibrinogen. *P < 0.05, **P < 0.01, ***P < 0.001, ****P < 0.0001, N = 6.

Twenty-four hours after rats underwent CLP treatment, platelet count (PLT) and prothrombin time (PT) were significantly decreased, while fibrinogen (FIB) levels were significantly elevated (**Figures 3A–C**). In contrast, no significant changes were observed in activated partial thromboplastin time (APTT) or thrombin time (TT) (**Figures 3D, E**). Under simulated weightlessness conditions, the platelet count in rats of the HU group significantly decreased (**Figure 3A**). Meanwhile, compared with the CLP group, rats in the HU+CLP group (subjected to CLP treatment for 24 hours under simulated weightlessness) exhibited significantly decreased platelet levels and significantly elevated PT and APTT levels (**Figures 3B, D**). This indicates that simulated weightlessness exacerbates coagulation dysfunction in the early stage of sepsis in rats.

### 3.4 Simulated weightlessness exacerbates multiorgan dysfunction in septic rats

**Figure 4:**
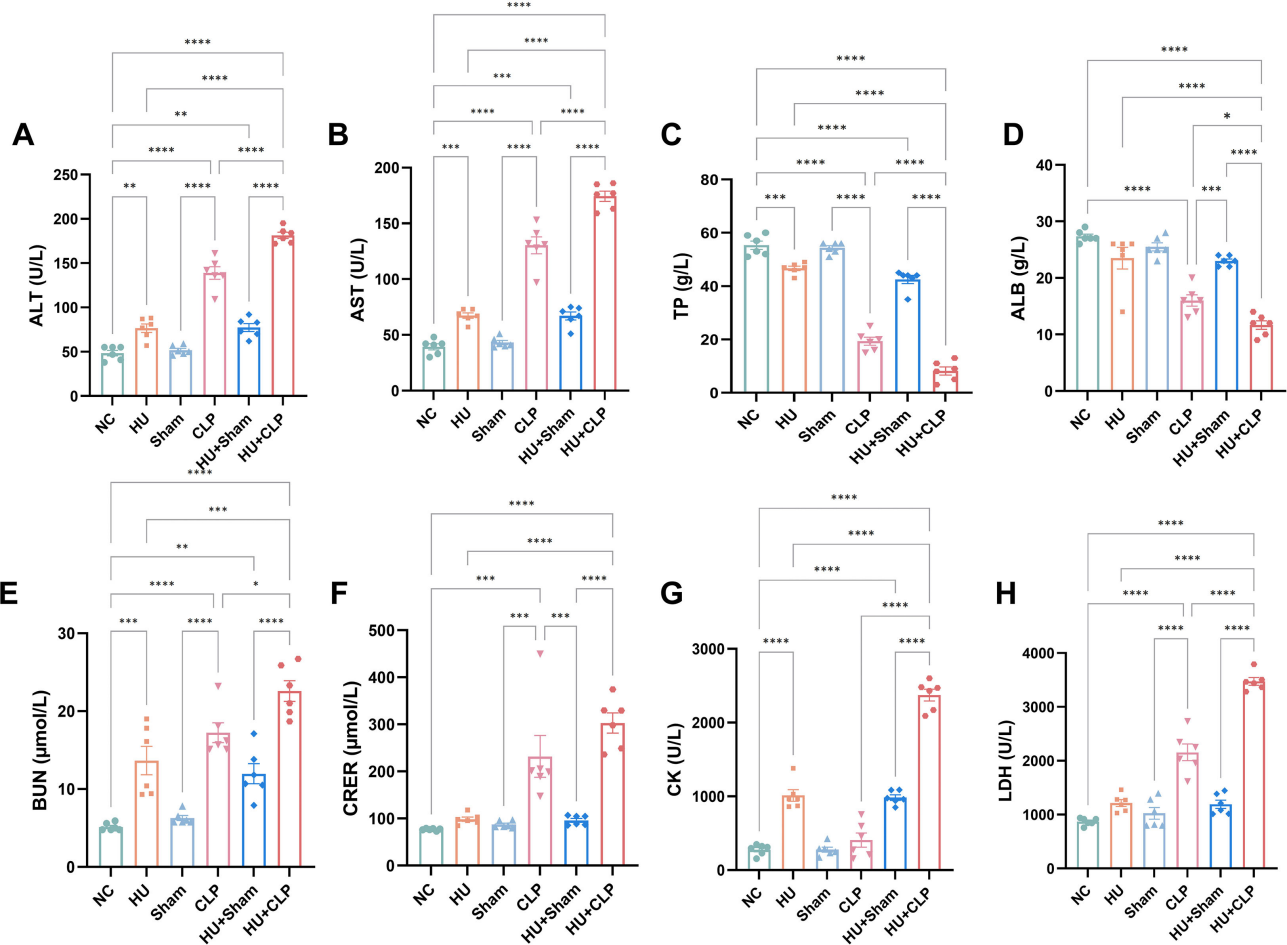
Biochemical index detection in serum of septic rats under weightlessness conditions. **(A)** Alanine aminotransferase; **(B)** Aspartate aminotransferase; **(C)** Total protein; **(D)** Albumin in rats; **(E)** Creatine kinase; **(F)** Lactate dehydrogenase; **(G)** Blood urea nitrogen (BUN) **(H)** Creatinine. *P < 0.05, **P < 0.01, ***P < 0.001,****P < 0.0001, N = 6.

Both simulated HU and CLP significantly increased serum levels of alanine aminotransferase (ALT) and aspartate aminotransferase (AST) while reducing total protein (TP) content in rats (**Figures 4A–C**). Additionally, serum albumin levels (ALB) were also decreased in CLP-induced septic rats (**Figure 4D**). In the HU+CLP group, serum ALT and AST levels were significantly higher than those in the CLP group, indicating that the weightlessness environment exacerbated hepatic dysfunction and hepatocellular injury in the early stage of sepsis (**Figures 4C, D**). Serum total protein and albumin levels in the HU+CLP group were significantly lower than those in the CLP group, reflecting aberrant hepatic protein anabolic metabolism. Under simulated weightlessness conditions, the serum urea nitrogen (BUN) levels in rats were significantly elevated (**Figure 4E**). CLP-induced septic rats also showed significantly increased serum BUN and creatinine (CRER) levels (**Figures 4E, F**). Notably, the BUN level in the HU+CLP group was significantly higher than that in the CLP group, further indicating impaired renal function and abnormal protein metabolism (**Figure 4E**). However, although the serum creatinine level in the HU+CLP group was higher than that in the CLP group, the difference did not reach statistical significance (**Figure 4F**). Meanwhile, the reductions in total protein and albumin shown in **Figures 4C, D** further support protein loss resulting from renal impairment. Simultaneously, To further investigate the impact of simulated weightlessness on cardiac function injury in septic rats, we measured serum creatine kinase (CK) and lactate dehydrogenase (LDH) levels. As shown in **Figures 4G, H**, after 29 days of simulated weightlessness, serum CK was elevated in rats, while serum LDH increased significantly 24 hours after CLP induction. Compared with the CLP-alone group, rats subjected to CLP under weightlessness conditions for 24 hours showed significantly higher levels of both serum CK and LDH. These results indicate that weightlessness exacerbates cardiac function impairment in the early stage of sepsis.

### 3.5 Simulated weightlessness exacerbates multi-organ pathological damage in septic rats

**Figure 5:**
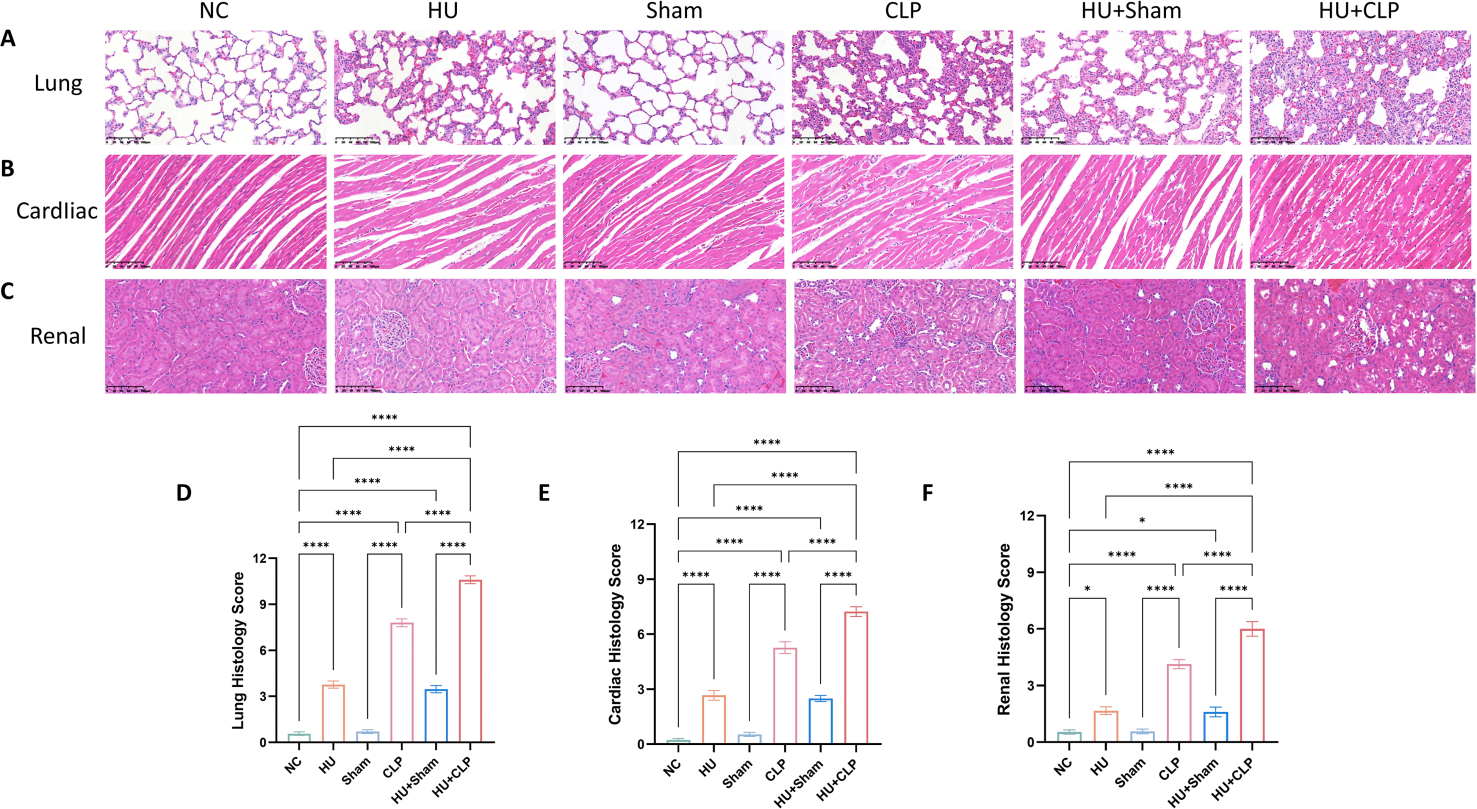
Histopathological staining of rat tissue sections. **(A)** H&E staining of rat lung tissue; **(B)** H&E staining of rat heart tissue; **(C)** H&E staining of rat kidney tissue; **(D)** Semi-quantitative analysis of H&E staining of lung tissue; **(E)** Semi-quantitative analysis of H&E staining of heart tissue; **(F)** Semi-quantitative analysis of H&E staining of kidney tissue. *P < 0.05, ****P < 0.0001, N = 6.

To further assess the pathological damage to rat organs, we collected the lungs, hearts, and kidneys from each group at 24 hours after CLP and performed H&E staining. As shown in **Figures 5A, D**, both the HU group and the CLP group exhibited a certain degree of pulmonary pathological damage, including inflammatory infiltration, alveolar wall thickening, and alveolar hemorrhage. In contrast, the HU+CLP group showed significantly more severe damage, indicating that simulated weightlessness significantly aggravated lung injury in septic rats. Compared with the HU and CLP groups, the HU+CLP group also displayed significantly greater inflammatory infiltration, myocardial fiber necrosis/lysis, and edema/hemorrhage, suggesting that simulated weightlessness significantly exacerbated cardiac injury in septic rats (**Figures 5B, E**). Similarly, the HU+CLP group showed significantly more severe inflammatory infiltration, glomerular/tubular injury, and edema/hemorrhage compared with the HU and CLP groups, indicating that simulated weightlessness significantly aggravated kidney injury in septic rats (**Figures 5C, F**). These findings suggest that simulated weightlessness may accelerate disease progression by exacerbating multi-organ pathological damage in septic rats.

## 4 Discussion

Weightlessness increases oxidative stress by enhancing the PI3K/Akt/Nrf2 signaling pathway in rats, thereby impairing mitochondrial function (Mu et al., 2025). Meanwhile, downregulation of the PI3K/Akt pathway, increased NF-κB expression, and F-actin depolymerization exacerbate endothelial injury (Kang et al., 2011). Spaceflight-induced processes such as oxidative and mitochondrial stress, autophagy, DNA damage repair, and alterations in telomere length directly or indirectly converge on the activation of inflammatory responses (Capri et al., 2023). Weightlessness affects immune system functions through multiple signaling pathways. Activation of the p38 MAPK-C/EBPβ signaling pathway upregulates arginase and IL−6 expression while downregulating IL−12B expression (Wang et al., 2015); the IL−6/STAT3 axis induces hepcidin expression, which in turn leads to iron metabolism disorders and inflammatory responses (Cavey et al., 2017). In a biological study by Garrett-Bakelman FE et al., a one-year mission on the International Space Station was found to alter the innate, adaptive, and NK cell-mediated immune systems (Garrett-Bakelman et al., 2019). A significant decrease in 11 bacterial species, including Akkermansia muciniphila and Eubacterium coprostanoligenes, has been observed in the intestines of weightless mice (Shi et al., 2017; Siddiqui et al., 2022). Exposure to weightlessness can lead to dysbiosis damage and intestinal epithelial (Zong et al., 2024). The pathophysiological changes caused by weightlessness not only compromise the health of astronauts but also elevate their risk of infection. With the continuous progress of manned space exploration, growing numbers of astronauts are being sent into space. The mechanisms of weightlessness-induced injury share similarities with core pathological features of sepsis, such as immunosuppression, imbalanced inflammatory responses, and coagulation disorders (Zhang and Ning, 2021; Liu et al., 2022; Srdić et al., 2024), which may accelerate deterioration and progression to sepsis following infection.

Four weeks of HU did not result in mortality in rats. The survival rate in the CLP group was 20%, while weightlessness reduced the survival rate of septic rats to 6.67%, suggesting that weightlessness−induced injury may accelerate the progression of sepsis (**Figure 1B**). Compared to the control group, significant changes were observed in the levels of HGB and PLT in the HU group, indicating blood cells abnormalities in the rats (**Figures 2E**, **3A**). The overall trend was generally consistent with previous studies (Rivera et al., 2006; Wilson et al., 2012). However, compared with the CLP group, the HU+CLP group exhibited significantly increased levels of MON, GRA, TGF−α, IL−6, and CRP, along with significantly decreased levels of HGB and IL−10 (**Figures 2C–E, G–J**). In the CLP−induced sepsis model, elevated neutrophil mitochondrial activity promotes the release of granule contents with endothelial disruptive capacity, while changes in granulocytes are likely associated with injury to organs such as the liver (Hayase et al., 2026; Zakrzewski et al., 2026). In CLP rats, these immune changes and the resulting organ injury are amplified and exacerbated under weightlessness, leading to more pronounced immune alterations. Additionally, our results showed that compared with the CLP alone group and the HU alone group, the combined HU+CLP group exhibited significant changes in biochemical function indices and pathological damage of the heart, liver, and kidneys, with more severe organ damage (**Figures 4**, **5**). Inflammatory responses play a central role in organ injury in the CLP model. Rel+ macrophages disrupt the homeostasis of the cardiac microenvironment through multiple signaling pathways, leading to cardiomyocyte dysfunction (Zhou et al., 2026). The YAP1/Nrf2 axis exacerbates oxidative stress and inflammatory responses, thereby aggravating liver injury (Zhang et al., 2025). Abnormal activation of the TLR4/NF-κB signaling pathway results in the release of large amounts of pro-inflammatory cytokines, causing damage to renal tubular epithelial cells (Hacım et al., 2026). Under the combined effect of weightlessness and sepsis, the important cause of organ injury is the significantly upregulated inflammatory response, along with the accompanying oxidative damage and mitochondrial damage.

In 2021, a case of internal jugular vein thrombosis requiring anticoagulant therapy was first reported (Limper et al., 2021). During spaceflight, changes in blood components, electrolyte imbalances, and other alterations may occur in the body. Meanwhile, the shift of body fluids toward the head leads to distension of the internal jugular vein and hemodynamic changes, including stasis and retrograde flow, resulting in a hypercoagulable state of the blood and an increased risk of thrombosis (White and Wenthe, 2023; Marshall-Goebel et al., 2024; Mayor et al., 2025). In the HU group, PLT decreased significantly, while TT and FIB levels showed an increasing trend, and APTT showed a decreasing trend, although these changes did not reach statistical significance (**Figures 3A, C–D**). This suggests that weightlessness leads to increased consumption or decreased production of platelets. Compared with the sepsis−only group, weightlessness significantly prolonged APTT and PT and significantly decreased PLT in septic rats. Certainly, subsequent studies may further explore the alterations in D−dimer or antithrombin III. The impact of sepsis on the coagulation system primarily involves a vicious cycle of vascular endothelial barrier disruption and immunothrombosis (Huang et al., 2026; Mohiuddin et al., 2026). These effects are present under weightlessness and develop rapidly after sepsis, accelerating coagulation dysfunction.

In this study, a rat model of sepsis under simulated weightlessness was initially established. While observing the mortality rate of septic rats under simulated weightless conditions, the effects of simulated weightlessness on the early development of sepsis in rats were evaluated using hematological, coagulation, and organ injury indicators. Without a doubt, this is a complex process, and the molecular mechanisms underlying its occurrence and development still require further in-depth investigation. However, the therapeutic efficacy of existing (e.g., Meropenem (Zhao et al., 2017), Piperacillin (Harris et al., 2018)) or emerging treatments (e.g., MSC (Zhai et al., 2024) (Shaw et al., 2021)) for weightlessness-sepsis remains to be confirmed. It is our hope that the present findings will offer a useful reference for researchers investigating the molecular underpinnings and therapeutic interventions of sepsis in the weightless environment, while also providing preclinical evidence to support the prevention and treatment of sepsis in astronauts during orbital missions. In the long term, we envisage that this work will facilitate the development of tailored strategies for the prevention and treatment of weightlessness−related sepsis, ultimately contributing to the health and safety of spaceflight personnel.

## Data Availability

The original contributions presented in the study are included in the article/supplementary material. Further inquiries can be directed to the corresponding authors.
